# Investigation of Brain Function-Related Myokine Secretion by Using Contractile 3D-Engineered Muscle

**DOI:** 10.3390/ijms23105723

**Published:** 2022-05-20

**Authors:** Takeshi Sugimoto, Tomohiro Nakamura, Sho Yokoyama, Toshia Fujisato, Satoshi Konishi, Takeshi Hashimoto

**Affiliations:** 1 Faculty of Sport and Health Science, Ritsumeikan University, Kusatsu 525-8577, Japan; hadano4249@gmail.com; 2 Division of Human Sciences, Faculty of Engineering, Osaka Institute of Technology, Ohmiya 535-8585, Japan; tomohiro.nakamura@oit.ac.jp; 3 Department of Mechanical Engineering, School of Engineering, Osaka Institute of Technology, Ohmiya 535-8585, Japan; sho.yokoyama@oit.ac.jp; 4 Graduate Course in Applied Chemistry, Environmental and Biomedical Engineering, Osaka Institute of Technology, Ohmiya 535-8585, Japan; toshiya.fujisato@oit.ac.jp; 5 Department of Mechanical Engineering, College of Science and Engineering, Ritsumeikan University, Kusatsu 525-8577, Japan; konishi@se.ritsumei.ac.jp

**Keywords:** tissue-engineered muscle, C2C12, myokine, electrical pulse stimulation, muscle contraction

## Abstract

Brain function-related myokines, such as lactate, irisin, and cathepsin B (CTSB), are upstream factors that control brain-derived neurotrophic factor (BDNF) expression and are secreted from skeletal muscle by exercise. However, whether irisin and CTSB are secreted by muscle contraction remains controversial. Three-dimensional (3D)-engineered muscle (3D-EM) may help determine whether skeletal muscle contraction leads to the secretion of irisin and CTSB, which has never been identified with the addition of drugs in conventional 2D muscle cell cultures. We aimed to investigate the effects of electrical pulse stimulation (EPS)-evoked muscle contraction on irisin and CTSB secretion in 3D-EM. The 3D-EM, which consisted of C2C12 myoblasts and type-1 collagen gel, was allowed to differentiate for 2 weeks and divided into the control and EPS groups. EPS was applied at 13 V, 66 Hz, and 2 msec for 3 h (on: 5 s/off: 5 s). Irisin and CTSB secretion into the culture medium was measured by Western blotting. Irisin secretion was significantly increased following EPS (*p* < 0.05). However, there was no significant difference in CTSB secretion between the two groups. The present study suggests that irisin may be a contractile muscle-derived myokine, but CTSB is not secreted by EPS-evoked muscle contractile stimulation in 3D-EM.

## 1. Introduction

Myokine, a bioactive substance secreted from skeletal muscle upon exercise stimulation [1], plays an important role as an upstream factor regulating the exercise-induced increase in brain-derived neurotrophic factor (BDNF) expression in the brain [2,3]. Given that BDNF promotes neurogenesis, synaptic plasticity, and cell survival [4] and improves cognitive function [5,6], BDNF regulation by myokines might be relevant for brain health. Lactate [7,8,9], fibronectin type III domain-containing protein 5 (FNDC5)/irisin [10], and cathepsin B (CTSB) [11] are myokines associated with brain function. Specifically, lactate is secreted from skeletal muscle by mechanical muscle contractile stimulation in both in vitro [12,13] and in vivo [8,14] studies. Secreted lactate is delivered into the brain through blood vessels by crossing the blood-brain barrier (BBB) [15,16], which increases BDNF expression in the brain [7]. However, previous studies have shown that irisin and CTSB in the blood pass through the BBB [11,17] and increase BDNF expression in the brain [11,18]; thus, a series of working hypotheses suggest that irisin and CTSB are secreted from skeletal muscle as myokines, which in turn increase brain BDNF expression [3]. However, whether irisin and CTSB are secreted from skeletal muscle (i.e., into the blood) by muscle contraction is fundamentally unclear. In fact, previous studies have not examined the effects of in vitro mechanical muscle contractile stimulation on irisin and CTSB secretion; thus, these relationships should be elucidated to understand the importance of “exercise” (i.e., working muscle) for the secretion of these myokines. Since acute exercise does not alter blood irisin and CTSB levels [19,20,21], whether these myokines are secreted by muscle contraction remains controversial [2,3].

Previous studies that investigated myokine (e.g., irisin and CTSB) expression and/or secretion used pharmacological stimulation (e.g., 5-aminoimidazole-4-carboxamide ribonucleotide: AICAR, free fatty acid: FFA) as exercise mimetics in two-dimensional (2D) muscle cell cultures such as C2C12 and L6 myocytes [11,22]. However, pharmacological stimulation can only partially represent the activation of intracellular signals that can occur during locomotion in vivo [23]. In contrast, electrical pulse stimulation (EPS) can lead to mechanical muscle contraction and mimic the overall intracellular signal activation to exercise [23]. Therefore, EPS may be suitable for the identification of contractile muscle-derived myokines.

Although previous studies used EPS in 2D muscle cell cultures [24,25], these cells are structurally different from in vivo skeletal muscle tissue [26,27]. However, three-dimensional (3D)-engineered muscle (3D-EM) is fabricated by conventional 2D muscle cell cultures along with extracellular matrix components, such as type I collagen [27]. Additionally, the previous study suggested that the 3D model has the alignment of differentiated myotubes in parallel with the long plane of the gel, whereas the 2D cultural model showed no such organization [27]. Indeed, we previously confirmed parallel myotube alignment in the 3D-EM [28]. Furthermore, 3D-EM is structurally composed of highly dense, parallel-orientated fascicle-like structures of myofibers [29], forming sarcomere structures similar to those found in living muscle [30]. Thus, a 3D-EM may more closely represent in vivo muscles. Importantly, some myokines show higher intramuscular expression in 3D-EM than in 2D muscle cell cultures [31], which may uncover findings that would be unclear in 2D muscle cell cultures. Moreover, the EPS-evoked muscle contractile force in 3D-EM is 10 times higher than that in 2D muscle cell cultures [30]. In addition, the 3D-EM can be cultured without being attached to the culture plate, thereby expanding the contraction range in the long axis direction and inducing greater contraction behavior than that of conventional 2D muscle cell cultures. Collectively, the 3D-EM may provide an advanced approach for elucidating whether skeletal muscle contraction leads to the secretion of brain function-related myokines, which have never been identified with the addition of drugs in conventional 2D muscle cell cultures. The purpose of the present study was to investigate the effects of EPS-evoked muscle contractile stimulation on irisin and CTSB secretion in 3D-EM. We hypothesized that EPS-evoked muscle contractile stimulation in 3D-EM can increase irisin and CTSB secretion.

## 2. Results

### 2.1. Skeletal Muscle Contractile Behavior in 3D-EM

To determine whether EPS promotes the muscle contractile response in 3D-EM, we photographed 3D-EM during EPS under a microscope (Supplementary material Video S1). As a result, the PDMS pillars tilted due to the strong contraction of the construct when EPS was applied, and the tilt quickly returned to normal when EPS application was stopped.

### 2.2. Changes in Metabolites in the Culture Medium

**Figure 1:**
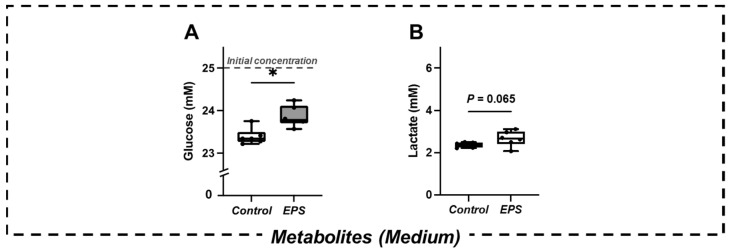
Changes in the concentration of metabolites ((**A**) glucose and (**B**) lactate) in the culture medium (*n* = 6). The initial concentration of glucose in the culture medium was 25 mM. Values are presented as the median (horizontal line in the box), range between 25th and 75th quartiles (box), and maximum and minimum values (whisker). * Significant difference between groups (*p* < 0.05).

The glucose concentration in the culture medium of the EPS group was significantly higher than that in the CON group (*p* < 0.05) (Figure 1A). The lactate concentration in the culture medium in the EPS group exhibited an increasing trend compared to that in the CON group (*p* = 0.065) (Figure 1B).

### 2.3. Changes in Intracellular Signaling and Myokine Expression

**Figure 2:**
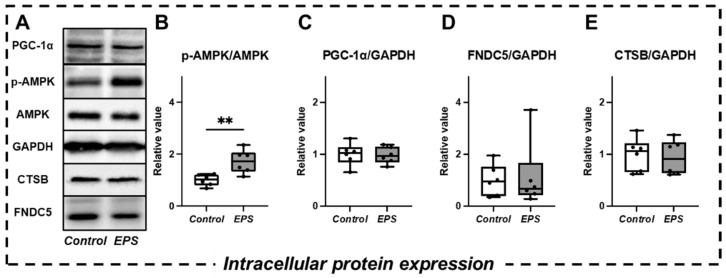
Comparison of intracellular protein expression in 3D-EM (**A**). Western blot analyses with anti-phosphorylated AMPK (**B**), PGC-1α (**C**), FNDC5 (**D**), and CTSB (**E**) were performed (*n* = 6). All protein expression levels were normalized to the GAPDH level. Proteins are expressed relative to the value of the control protein levels (relative value). Values are presented as the median (horizontal line in the box), range between the 25th and 75th quartiles (box), and maximum and minimum values (whisker). At least two independent experiments were performed. ** Significant difference between groups (*p* < 0.01).

The previous study suggested that EPS increases PGC-1α expression and AMPK phosphorylation activity, which regulates Irisin [10] and CTSB [11] secretion, respectively [32]. Therefore, these markers may not only demonstrate muscle contractile activity during exercise in humans and the corresponding signaling responses in 2D muscle cell cultures [13] but also become factors that explain the mechanism of brain function-related myokine secretions. We observed significant activation of intracellular AMPK phosphorylation in the EPS group compared to the control group (*p* < 0.01) (Figure 2B). In contrast, intracellular peroxisome proliferator-activated receptor-γ coactivator 1α (PGC-1α) expression and the expression of various myokines (FNDC5, CTSB) were not significantly different between the two groups (Figure 2C–E).

### 2.4. Changes in Extracellular Protein Expression and Myokine Secretion in the Culture Medium

**Figure 3:**
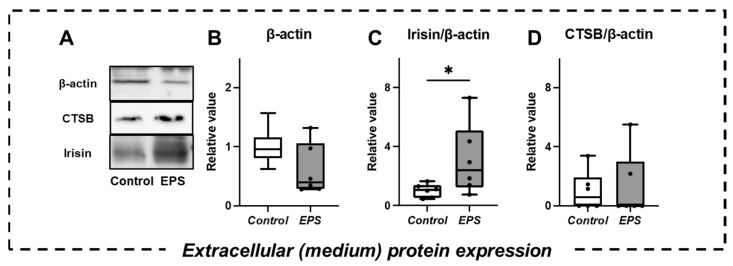
Comparison of extracellular protein expression in culture medium (**A**). Western blot analyses with anti-β-actin (**B**), irisin (**C**), and CTSB (**D**) were performed (*n* = 6). Irisin and CTSB expression levels were normalized to the β-actin level. Proteins are expressed relative to the value of the control protein levels (relative value). Values are presented as the median (horizontal line in the box), range between the 25th and 75th quartiles (box), and maximum and minimum values (whisker). At least two independent experiments were performed. * Significant difference between groups (*p* < 0.05).

There was no significant difference in β-actin protein expression between the two groups (Figure 3B), suggesting that EPS did not elicit cell damage [33]. Irisin secretion was significantly increased in the EPS group compared with the control group (*p* < 0.05) (Figure 3C). However, no significant difference in CTSB secretion was confirmed between the two groups (Figure 3D).

## 3. Discussion

The present study demonstrated that EPS-evoked muscle contractile stimulation in 3D-EM increased the secretion of irisin but not CTSB. Therefore, in terms of muscle-brain crosstalk, irisin, as a myokine, may be an important contributor to the exercise-induced increase in BDNF expression.

Irisin is cleaved from FNDC5, a transmembrane precursor protein expressed in skeletal muscle under the control of PGC-1α [10]. Acute exercise elevated blood irisin levels in humans [34]. However, since blood level might reflect not only secretions of skeletal muscle but also that of other organs, whether irisin was secreted by contractile muscle was unclear. The present study demonstrated for the first time that EPS-evoked muscle contractile stimulation in 3D-EM increases irisin secretion, suggesting that irisin may be a contractile muscle-derived myokine.

In contrast, EPS-evoked muscle contractile stimulation in 3D-EM did not alter intracellular FNDC5 expression, which is regulated by PGC-1α, and increased extracellular secretion of irisin is accompanied by a PGC-1α-dependent increase in intracellular FNDC5 [10]. Given these inconsistent results, the exercise-induced increase in irisin secretion may be due to mechanical muscle contractile stimulation itself but not a change in intracellular metabolism.

CTSB secretion was not increased by EPS-evoked muscle contractile stimulation, suggesting that CTSB might not be a contractile muscle-derived myokine. A previous study reported that the addition of AICAR to 2D muscle cell cultures increases the concentration of CTSB in the culture medium [11], suggesting the importance of increased AMPK phosphorylation on skeletal muscle CTSB secretion. In the present study, although EPS-evoked muscle contractile stimulation in 3D-EM increased AMPK phosphorylation, CTSB secretion was not altered. Given that acute resistance exercise [19] and high-intensity interval exercise [20] did not change blood CTSB levels in humans, CTSB secretion may not be altered by acute exercise (i.e., muscle contraction). Previously, the CTSB concentration in the culture medium increased after the addition of the AMPK agonist AICAR for 6 h [11], which might elicit prolonged AMPK phosphorylation compared with our EPS application for 3 h. In addition, given that changes in maximal oxygen uptake and blood CTSB concentrations are positively correlated after a 4-month aerobic training intervention in humans [11], the elevation of CTSB secretion may respond to long-term AMPK activation. Therefore, CTSB may not be a myokine derived from acute muscle contraction but may be a myokine secreted homeostatically by skeletal muscle due to chronic training-induced metabolic changes. However, some studies have shown that blood CTSB levels did not change after chronic aerobic and resistance training interventions [35,36]; thus, further studies will be needed to elucidate the detailed secretion mechanism of CTSB.

The present study revealed that EPS-evoked muscle contractile stimulation in 3D-EM tends to increase the concentration of lactate in the culture medium, which is consistent with previous studies [12,13], suggesting muscle contraction enhanced glycolytic metabolism in skeletal muscle cells [37]. On the other hand, although previous studies have consistently shown that EPS-evoked muscle contractile stimulation in 2D muscle cell cultures decreases the glucose concentration in the culture medium [13,37], the EPS group in the present study showed significantly higher levels than the control group. Although the detailed factors are unknown, the changes in glucose uptake and muscle glycogen content may be influenced by the difference between 2D and 3D cultures. To explain the inconsistency with previous 2D muscle cell culture studies, further studies will be needed to measure the amount of glycogen in 3D-EM.

Recently, from the viewpoint of animal ethics issues [26,38], the demand for the use of novel “animal-free” 3D-culture models based on tissue engineering has increased in the fields of not only regenerative medicine [39] but also the health sciences [40]. Importantly, previous studies have only examined intracellular myokine expression [31,41], and we first established a platform to verify the secretion of myokines in 3D-EM, which is more mature than 2D muscle cell cultures (higher myosin heavy chain protein expression, data not shown). Indeed, numerous myokines are produced in response to exercise and function as “real polypills” by circulating in the body to target tissues and organs [42]; however, we have focused on brain health-related myokines given our previous study showing improved cognitive function in response to acute exercise [8]. Regarding the physiological properties of irisin, for instance, its beneficial health potential in the maintenance of a variety of tissues and organs has been suggested [43]. Thus, exploring the effects of myokines on multimodal EPS would help develop exercise prescriptions for health benefits. Moreover, given that admission of drug and/or nutritional substrate relates to secretions of myokine [11,44], further multilateral research on exercise-induced secretion factors such as myokines in 3D-EM will not only help to demonstrate the importance of exercise but may also contribute to drug and/or nutrition development for patients with chronic diseases who have difficulty implementing exercise.

As an important perspective, a previous study used inhibitors that block each physiological factor (e.g., N-benzyl-p-toluene sulfonamide and EGTA) to investigate whether muscle contraction itself or the accompanying calcium influx is important as a determinant of the secretion of the major myokine interleukin-6 [24]. Therefore, further study is needed to elucidate the relationship between irisin secretion and mechanical muscle contractile stimulation. In addition, since different EPS conditions (e.g., intensity, frequency, duration, contraction type) are associated with altered cellular responses [32], future studies examining myokine secretion under different EPS conditions will be needed.

## 4. Materials and Methods

### 4.1. Molding Devices and Artificial Tendon

**Figure 4:**
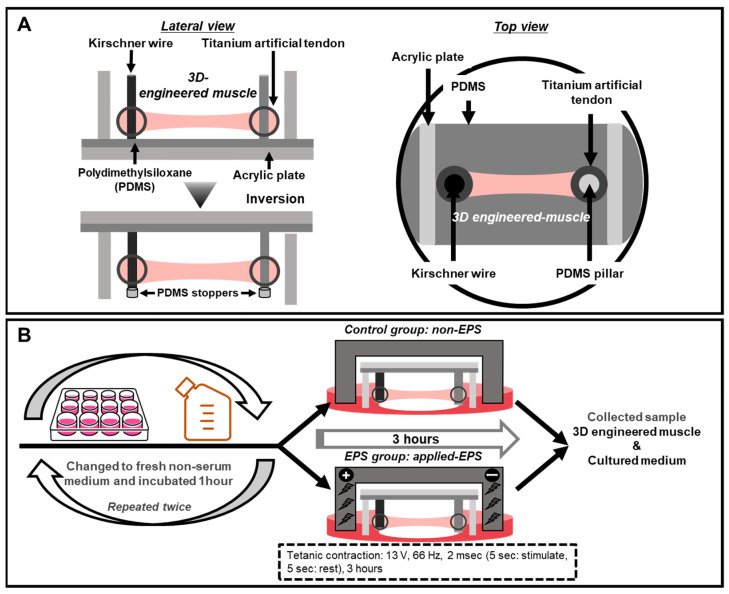
(**A**) A titanium artificial tendon was inserted into each of the Kirschner wires and a PDMS pillar. C2C12 myoblasts were embedded in Cell Matrix Type 1-A at a density of 1.5 × 10^7^ cells/mL. A 100 µL gelled cell suspension was seeded between the two artificial tendons. After 10 days of differentiation, the device was inverted and cultured continuously for a total of 14 days. (**B**) Experimental cell culture and EPS procedure. The 3D-EM differentiated for 14 days was washed twice with PBS. The serum-free medium was replaced with PBS and incubated for 1 h, and then, this process was conducted twice. The 3D-EM was subjected to tetanic contractile stimulation. Immediately after applying EPS, the 3D-EM and medium samples were collected.

The device for culturing skeletal muscle was partially modified from that used in previous studies. Two fixing points of the previous device were not deformed [30,31], whereas the device in the present study had pins on one side made of PDMS [45], which can be deformed and inclined, thereby allowing a wider contraction range for the evaluation of myokine secretion. We used a molded titanium scaffold (Zellez ™; Hi-Lex Corporation, Hyogo, Japan) as the artificial tendon, which was shown in a previous study [28]. The titanium artificial tendon was attached to the Kirschner wire and the PDMS pillar (Figure 4A).

### 4.2. C2C12 Cell Culture

We cultured C2C12 skeletal muscle cells (American Type Culture Collection, Manassas, VA, USA). The C2C12 myoblasts were grown in growth medium (GM) consisting of high-glucose (4.5 g glucose/L) Dulbecco’s modified Eagle’s medium (DMEM; Nacalai Tesque, Kyoto, Japan) with 10% fetal bovine serum (FBS; Sigma-Aldrich, St. Louis, MO, USA), 100 U/mL penicillin, and 100 μg/mL streptomycin (PS; Nacalai Tesque) [46,47,48,49]. The GM was changed every two days.

### 4.3. Three-Dimensional Culture in Collagen Gel

The 3D-EM was constructed using C2C12 cells based on previous studies [28,30,31]. C2C12 cells were embedded in a cold type-1 collagen gel solution (Cellmatrix; Nitta Gelatin, Osaka, Japan) at a density of 1.5 × 10^7^ cells/mL. A 100-µL cold suspension of C2C12 cells was added between and on the surface of the two artificial tendons (Figure 4A). The constructs were placed in GM and cultured for two days. The GM was replaced with a differentiation medium (DM) consisting of high-glucose (4.5 g glucose/L) DMEM with 7% horse serum (HS; Thermo Fisher Scientific, Tokyo, Japan) and PS and cultured for 14 days. After 10 days of differentiation, the device was inverted and cultured to make it easier to photograph under a microscope. In addition, stoppers made of PDMS were attached to the ends of the Kirschner wire to prevent the artificial tendons from detaching from the pins (Figure 4A).

### 4.4. Electrical Pulse Stimulation (EPS)

We divided 3D-EM into two groups (control and EPS). The 3D-EM was differentiated for 14 days in a six-well dish and placed in a chamber for EPS (C-Dish; Ion Optix, Westwood, MA, USA). EPS was applied to the 3D-EM in a C-Dish using an electrical stimulation generator C-Pace (Ion Optix, Westwood, MA, USA). We changed from DM to DMEM with PS (serum-free medium) before incubating 3D-EM for an hour. In both groups, to minimize the myokine secretion effect by medium replacements [24], we repeated this process twice. The 3D-EM was subjected to EPS for 3 h under tetanus contractile stimulation conditions (13 V, 66 Hz, 2 ms, on: 5 s/off: 5 s) [13] (Figure 4B).

### 4.5. Metabolite Measurement

We measured the lactate concentration in the culture medium by an external biosensor (BF-7, Oji Scientific Instruments, Hyogo, Japan), which can detect specific metabolites with the corresponding kit. After contractile stimulation, the culture medium was collected, and the samples with phenol red were directly applied to the external biosensor [50].

### 4.6. Western Blot Analysis

Western blotting was performed essentially as described previously [28]. Briefly, the 3D-EM was homogenized in RIPA buffer with protease and phosphatase inhibitors [28]. The culture medium was centrifuged at 3500 rpm for 120 min at 4 °C to concentrate proteins using 3 kDa cutoff centrifugal filters (Millipore, Billerica, MA, USA). Cell lysates (15 μg) and concentrated medium of equal volume were subjected to SDS-polyacrylamide gel electrophoresis (SDS-PAGE), and the separated proteins were transferred to polyvinylidene difluoride membranes. Target proteins were probed with antibodies against peroxisome proliferator-activated receptor gamma coactivator 1-alpha (PGC-1α) (1:200; Santa Cruz; Dallas, TX, USA; sc-517380), AMPK (1:1000; Cell Signaling Technology, Danvers, MA, USA; #2793), phosphorylated-AMPK (Thr172) (1:500; Cell Signaling Technology, Danvers, MA, USA; #25350, CTSB (1:200; Abcam, Cambridge, MA, USA; ab58802), FNDC5/Irisin (1:1000; Abcam, Cambridge, MA, USA; ab174833), Glyceraldehyde-3-phosphate dehydrogenase (GAPDH) (1:10,000; Sigma-Aldrich, St. Louis, MO, USA; G9545), β-actin (1:500; Cell Signaling Technology, Danvers, MA, USA; #4967). Chemiluminescence was quantified using the Luminata Forte Western HRP Substrate (Millipore), followed by detection with the FUSION FX7 EDGE (Vilber Lourmat, Collégien, France). ImageJ software was used (National Institutes of Health, Bethesda, MD, USA) to quantify the band intensities and protein expression. Glyceraldehyde-3-phosphate dehydrogenase (GAPDH) and β-actin were used as intracellular and extracellular (medium) loading controls, respectively. The previous study has used the expression of β-actin, but not GAPDH, in the culture medium to normalize myokine secretion [24]. Therefore, we used two different housekeeping proteins in the present study.

### 4.7. Statistical Analysis

The data are shown as the median (interquartile range, IQR), and the Mann–Whitney U test, which does not assume a normal distribution, was used for comparisons between groups as a test of significance. Graph Pad Prism version 9.2.0 (283) was used for statistical processing of all data and the creation of diagrams. The significance level was set to *p* < 0.05.

## 5. Conclusions

It remained controversial whether brain function-related myokines such as irisin and CTSB are secreted by muscle contraction. By using 3D-EM, which may more closely represent in vivo skeletal muscles, the present study suggests that irisin may be a contractile muscle-derived myokine, but CTSB is not secreted by EPS-evoked muscle contractile stimulation. This 3D-EM is not only an alternative to animal experiments but also a novel skeletal muscle cell culture model that could accelerate future myokine research.

## Data Availability

Not applicable.

## Supplementary Materials

The following supporting information can be downloaded at: https://www.mdpi.com/article/10.3390/ijms23105723/s1.
